# Picornavirus 3C Proteins Intervene in Host Cell Processes through Proteolysis and Interactions with RNA

**DOI:** 10.3390/v15122413

**Published:** 2023-12-12

**Authors:** Somnath Mondal, Gisoo Sarvari, David D. Boehr

**Affiliations:** Department of Chemistry, The Pennsylvania State University, University Park, PA 16802, USA

**Keywords:** picornavirus, 3C protease, virus replication, host cell defense, protein structure, protein dynamics

## Abstract

The *Picornaviridae* family comprises a large group of non-enveloped viruses with enormous impact on human and animal health. The picornaviral genome contains one open reading frame encoding a single polyprotein that can be processed by viral proteases. The picornaviral 3C proteases share similar three-dimensional structures and play a significant role in the viral life cycle and virus–host interactions. Picornaviral 3C proteins also have conserved RNA-binding activities that contribute to the assembly of the viral RNA replication complex. The 3C protease is important for regulating the host cell response through the cleavage of critical host cell proteins, acting to selectively ‘hijack’ host factors involved in gene expression, promoting picornavirus replication, and inactivating key factors in innate immunity signaling pathways. The protease and RNA-binding activities of 3C are involved in viral polyprotein processing and the initiation of viral RNA synthesis. Most importantly, 3C modifies critical molecules in host organelles and maintains virus infection by subtly subverting host cell death through the blocking of transcription, translation, and nucleocytoplasmic trafficking to modulate cell physiology for viral replication. Here, we discuss the molecular mechanisms through which 3C mediates physiological processes involved in promoting virus infection, replication, and release.

## 1. Introduction

The positive-strand RNA viruses from *Picornaviridae* have tremendous medical, veterinary, and agricultural importance [1,2,3,4]. These viruses affect the digestive and respiratory systems, skin, liver, and heart in humans and animals. This family of monocistronic positive-strand RNA viruses consists of 63 genera and 285 different virus species, including enterovirus, rhinovirus, hepatovirus, and cardiovirus [5]. 

**Figure 1:**
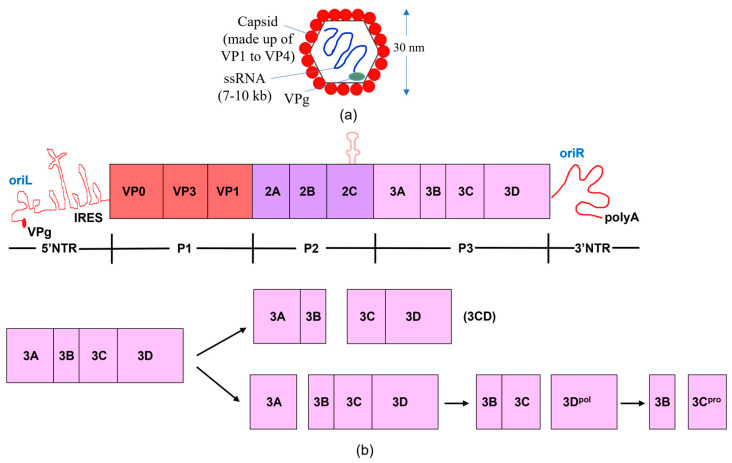
The genome and proteome of poliovirus. (**a**) Structure of poliovirus. (**b**) The processing of polyproteins and the poliovirus genome are shown schematically. The poly (A) tail, broad open reading frame, and 3′ and 5′ non-translated regions (NTR) comprise the poliovirus genome. The type II internal ribosome entry site (IRES) and a cloverleaf structure comprise the 5′NTR. The virus-encoded 3B (VPg) protein is attached to the 5′ end of the RNA. The viral genome encodes a single polyprotein; the P1 region encodes the structural proteins of the virus, while the P2 and P3 portions encode the non-structural proteins. The P3 region is cleaved by viral proteases via two main pathways, resulting in the production of the 3C and 3D proteins, either separately or collectively as 3CD. The RNA-dependent RNA polymerase 3D replicates the viral RNA, while 3C, a protease, cleaves at specific, conserved motifs located within flexible linkers that separate discrete proteins within the viral polyprotein. Because 3CD lacks polymerase activity and has a unique protease specificity that enables it to cleave the P1 capsid region differently than 3C, the functional activity changes when the 3C and 3D proteins are combined to produce 3CD [27,28,29,30].

The picornaviral virion and genome structures are highly conserved in nature. The genome consists of one open reading frame coding for a single polyprotein that can be targeted by viral proteases [3,6,7,8]. The P1 region encodes for the capsid proteins of the virus, while the P2 and P3 portions encode the non-structural proteins (Figure 1). The genome incorporates an open reading frame (ORF), a 5′ untranslated region (5′ UTR), and a 3′ untranslated region (3′ UTR) [9,10,11,12]. There is a polyadenylated (poly(A)) tail in the 3′ UTR. These viruses are non-enveloped and confine single-stranded RNA (length: 7–10 kb) with an icosahedral symmetric structure, having a diameter of about 30 nm (Figure 1) [13,14]. A covalently attached viral nucleotidylylated protein that caps a genome (VPg or, 3B) is present at the 5′ end of 5′ UTR [9]. The RNA genome carries an internal ribosome entry site (IRES) segment within the 5′ UTR to direct viral protein synthesis while employing ribosomes and other host factors [15,16].

The monocistronic single-strand RNA encodes only one polyprotein that is proteolytically cleaved by virally encoded proteases into several mature proteins, varying upon the genus [17,18]. The N-terminal region of the polyprotein is joined to a leader protein (L) in some viruses from various genera, including cardiovirus and aphthovirus [19,20,21,22]. The co- and post-translation processing of the polyprotein results in the expression of structural proteins 1A to 1D (VP4, VP2, VP3 and VP1), non-structural proteins (2A, 2B, 2C, 3A, 3B, 3C, 3D), or their intermediates (e.g., 3ABCD, 3ABC, 3BCD and 3CD) [1,23,24]. The 3C or its 3CD precursor are predominantly responsible for the proteolytic transformation of the polyprotein [25,26].

Almost fifty years ago, proteolytic processing was demonstrated to be crucial for picornavirus capsid protein synthesis and the construction of virions [27,28,29,30]. The 3C protein is recruited to perform this activity [31]. The 3C protein participates in the processing of polypeptides, binding of RNA, initialization of protein-priming of RNA synthesis, and viral translation to replication steps [1,3,19]. Antiviral therapeutic targets for 3C and similar viral proteases have been already identified [32]. In this Review, we emphasize various functional roles of 3C, including in the regulation of RNA replication, transcription, and translation, as well as its ability to inhibit nucleocytoplasmic transport and trigger apoptosis. Considering all these vast functions of 3C, this protease acts as an important virulence factor of picornaviruses [3].

## 2. Structure and Functions of 3C

### 2.1. Catalytic and RNA-Binding Sites

The 3C protease has a catalytic triad resembling those of serine proteases belonging to the trypsin-like family, although the catalytic nucleophile is a cysteine (Cys) residue rather than a serine (Ser) [33,34]. The X-ray crystal structure of human rhinovirus (3C) was determined nearly three decades ago (PDB ID:1CQQ) [35], followed by structure determination of 3C from other viruses including poliovirus (PV; PDB ID:1L1N) [36], hepatitis A virus (HAV; PDB ID:1QA7) [37], foot-and-mouth disease virus (FMDV; PDB ID:2BHG) [38,39], coxsackievirus B (CVB; PDB ID:2ZU1) [40,41,42], and enterovirus 71 (EV71; PDB ID:3OSY) [43]. All these 3C proteins are highly structurally similar.

Protein sequence similarities and phylogenetic relationships were previously established using the maximum likelihood method [44]. The catalytic triad, consisting of a Cys, His, and either Glu or Asp, was found to be highly conserved among the 3C proteases. The N-terminal alpha-helix, an important structural element involved in lipid membrane interactions, and the RNA binding region are not so conserved among picornaviral 3C proteases [45,46]. Six primary classes of 3C proteases from picornaviruses were identified by the phylogenetic study, in which the 3C proteases exhibited 38–45% amino acid similarity, a higher degree of sequence similarity than that observed in other picornaviruses.

**Figure 2:**
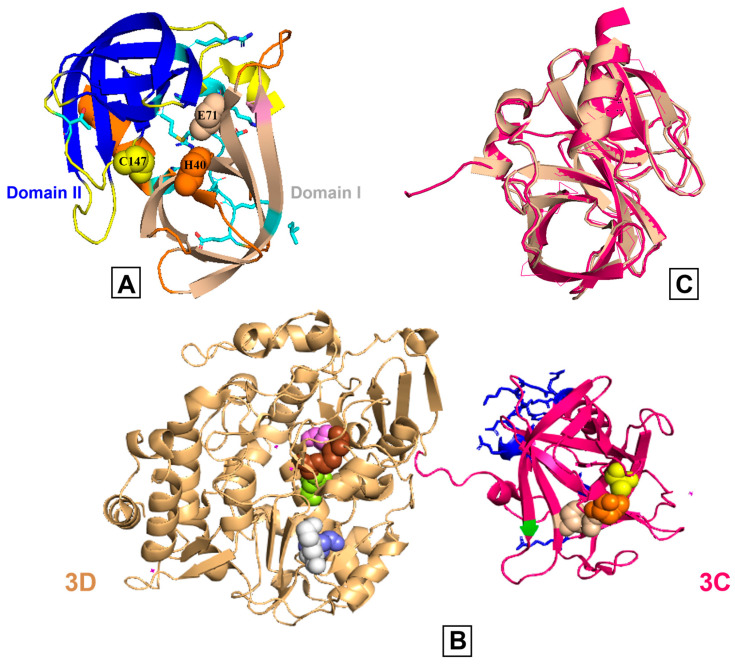
Comparison of poliovirus 3C and 3CD proteins. (**A**) The poliovirus 3C protease X-ray crystal structure (PDB: 1L1N) is shown, and the β-barrels of domains I and II are represented in wheat and blue colors, respectively. The catalytic triad (His40 (orange), Glu71 (light wheat), Cys147 (yellow)) is shown in spheres and the substrate pockets are in different colors. Side chains are colored by heteroatoms and the RNA binding region (E81-H89) is highlighted. (**B**) The X-ray crystal structure of poliovirus 3CD (PDB: 2IJD); the wheat and pink colors represent 3D and 3C, respectively. The active site residues are shown on both subdomains using spheres (3C: His40 (orange), Glu71 (light wheat), Cys147 (yellow); 3D, starting at the first residue of the 3D domain: Arg174 (brown), Asp233 (slate), Ser288 (violet), Asn297 (lemon), Lys359 (white)). The RNA binding region of 3C is colored blue. (**C**) The 3C region of 3C (pink) and 3CD (wheat) are structurally comparable as evidenced by an overlay with an RMSD value of 0.46 angstroms.

Our own research is primarily focused on PV 3C [47,48,49]. The PV 3C protein structure has a traditional trypsin-like fold (Figure 2) [36,50], despite having less than 10% sequence identity with the trypsin protease family. It includes six β-sheets that fold into two β-barrel subdomains arranged at right angles [36,51]. The surface groove connecting these subdomains serves as the substrate-binding site, housing the central catalytic triad in the active site [36]. The 3C proteins from HRV [35], PV [36], CVB [41], and EV71 [43] have a Glu as part of the catalytic triad, whereas in HAV and FMDV it is an Asp [37,38]. The first member of the catalytic triad, the absolutely conserved Cys residue, serves as the catalytic nucleophile, which is then aided by the His general base and the carboxylate group of either the Asp or Glu residue. Despite the structural similarity to Ser proteases, mutagenesis of the active site Cys for a Ser gives an enzyme with a drastically reduced activity. Caspases also employ a Cys/His diad, but appear to be much more active than 3C [37]. As the third component of the triad (Glu or Asp) is not rigorously conserved across all 3C proteases and the side chains tend to point away from the active site His, its necessity to catalyze is not as clear.

The substrate-binding capsule is formed by a surface groove that sits between two β-barrel domains. The β-ribbons and substrates form a strong association once they are bound, enhancing catalytic effectiveness [34,36,43]. The 3C groove ensures accurate substrate binding, with the overlying β-strands directing the configuration and depth of the active-site region [36]. The X-ray crystal structure shows that the β-barrel adopts a flexible conformation to increase the likelihood of substrate identification when a substrate is absent [7,36,37,43,52]. The flexible loop that comes before the catalytic Cys most likely changes conformation in response to substrate binding [36]. The groove of 3C determines its substrate specificity, as the β-ribbon above it influences the shape and depth of the active site. In EV71 3C, Gly123 and His133, two significant residues (not part of the catalytic triad) near the β-ribbon base, function as hinges to control the ribbon inherent flexibility. Studies using structure-guided mutagenesis have demonstrated the significance of the hinge residues for the proteolytic function of EV71 3C [43,53].

### 2.2. Viral Polyprotein Processing and Substrate Recognition

Viral proteins are produced by the picornavirus family via the polyprotein processing. The majority of cleavage sites, including at the VP2–VP3, VP3–VP1, 2B–2C, 2C–3A, 3A–3B, 3B–3C, and 3C–3D junctions [18,28], (see Figure 1), are processed by 3C, with four exceptions: L–VP4, VP4–VP2, P1–2A, and 2A–2B [54,55]. For example, the leader protein (L) in aphthoviruses and erboviruses releases itself from VP0 [1]. In some picornaviruses (i.e., aphthoviruses, cardioviruses), ribosome skipping at the Asn–Pro–Gly–Pro site causes cleavage of 2A–2B. Almost all secondary cleavages in picornaviruses are caused by 3C [1]. Only 3C and the 3CD precursor are needed to accomplish all cleavages in the case of the Aichi virus, as L and 2A^pro^ lack proteolytic activity. Studies on enteroviruses and apthoviruses have demonstrated that 3CD^pro^ can cleave at VP2–VP3 and VP3–P1 more effectively than 3C [56,57]. SVV 3C protease cleaves the P1 polypeptide to provide VP0, VP1, and VP3 [58]. Most 3C proteases cleave at single sites such as (Q|G), (Q|S), (Q|R), (E|Q), and (E|G) [7] (see Table 1 for example).

Crystal structure analysis has provided some insights into substrate recognition. For poliovirus 3C, the specificity pockets are clearly delineated, and modelling investigations help explain the established substrate specificity [36]. For FMDV 3C, the distinct substrate preferences for specific residue types, as well as the relative promiscuity, are well explained by the structure of the enzyme–peptide complex [70]. For instance, a similar peptide binding mechanism is revealed by crystallographic analysis of the complex containing a modified VP1–2A peptide (APAKE|LLNFD) with a Gln-to-Glu substitution [70]. This also explains why 3C can cleave sequences containing either P1–Gln or P1–Glu.

In contrast, 3C EV71 takes on an unusually open conformation in which the active site is highly exposed to solvent because of its open β-ribbon shape. There are insufficient electron densities to identify the conformations of the active site, and none to define the side chain conformation of catalytic Glu71 [53]. This inherent flexibility may be important in recognizing diverse cleavage sites observed in virus and host protein targets.

### 2.3. 3C(D) Also Interacts with Virus Replication Membranes

How viral proteins specifically target genome replication sites has been a persistent question in the field of virology [71,72]. Virus infection changes the lipid and protein composition relative to membranes found in uninfected cells [73,74,75]. The field has long known that these RNA viruses manipulate host cell membranes to create specific replication sites [22], but only in the past decade or so has it been understood how/why virus proteins are relocalized to these replication membranes.

RNA viruses have been shown to induce the production of a lipid called phosphatidylinositol 4-phosphate (PI4P) in host cells. In 2010, Altan-Bonnet and colleagues found that PI4P was produced during picornavirus infection and associated with regions of genome replication [76]. The study revealed that lipids are vital for RNA replication, with viruses 3D RNA polymerase binding to specific lipids such as PI4P [76,77]. It is well known that phosphatidylinositol phosphate phospholipids (PIPs) serve as a type of “zip code” for protein sorting to the appropriate subcellular compartment [49,78,79]. For instance, the pleckstrin homology (PH) domain from phospholipase C is the most extensively studied PIP-binding determinant, which interacts with phosphatidylinositol 4,5-bisphosphate (PI(4,5)P2) [80,81,82]. These domains support latent enzymatic activity until activated by the proper PIP, limiting enzyme activation to the appropriate subcellular compartment [83,84,85,86]. These characteristics are ideal for a virus to direct its enzymes and proteins to the proper locations and regulate their functions in those areas.

**Figure 3:**
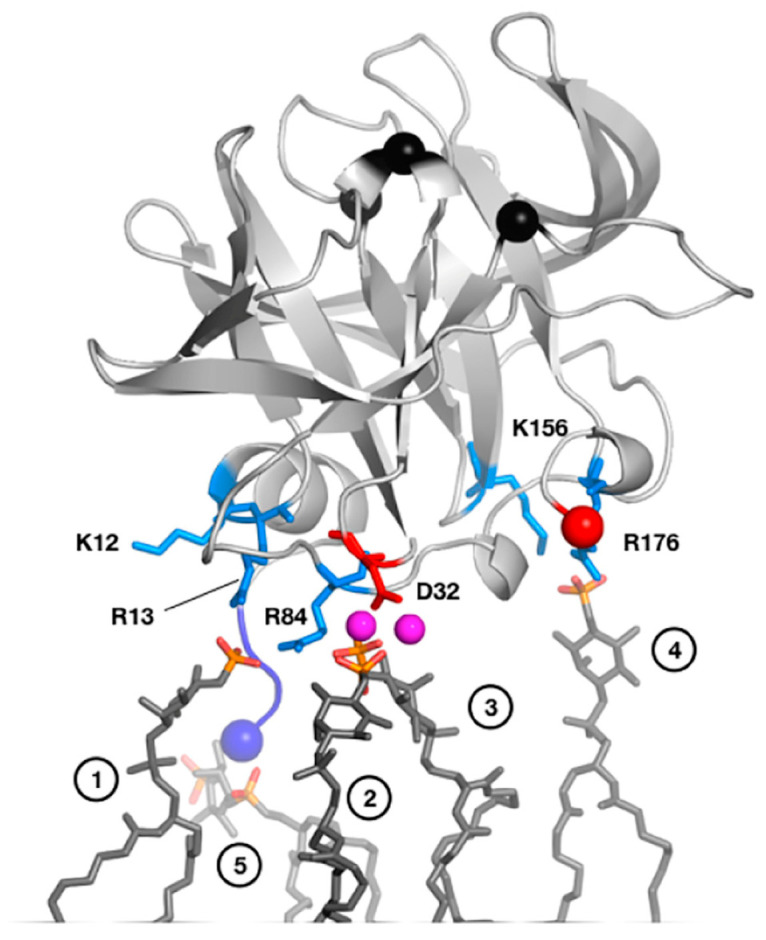
MD-derived model of PV 3C interactions with a PI4P-containing lipid membrane. Residues involved in interactions include Arg13 in the N-terminal alpha helix and Arg84 in the RNA-binding region. NMR studies with short-chain, soluble PI4P lipids largely confirm this model. In this model, PV 3C interacts with five clustered PI4P lipid molecules as shown in dark grey sticks with phosphates colored orange (phosphorus) and red (oxygen). The following specific interactions are listed, with numbers in parenthesis denoting the panel’s head group: (1) R13 and R84; (2) D32, mediated by sodium ions; (3) D32 mediated by sodium; (4) K156 and R176; and (5) α-amino group of G1. Reprinted from Structure, 25(12), D. Shengjuler, Y.M. Chan, S. Sun, I. Moustafa, Z.-L. Li, D.W. Gohara, M. Buck, P.S. Cremer, D.D. Boehr, C.E. Cameron. The RNA-binding site of poliovirus 3C protein doubles as a phosphoinositide-binding domain, 1875–1886, Copyright (2017) with permission from Elsevier.

PIP lipids found on the cell membrane can be used by picornaviruses to control viral RNA replication. PV 3C binds PI4P through its dynamic N-terminal α-helix, which competes with RNA binding due to the overlap between the PIP- and RNA-binding sites [49]. PIPs may therefore be used by picornaviruses to control viral RNA replication [76]. NMR and MD simulations have helped to delineate the PIP-binding site (Figure 3) [49].

The roles of 3C in the viral replication cycle may be better understood with further research into the structural characteristics and functional significance of PV 3C and its interactions with host cell membranes. The remaining part of the review emphasizes the ability of picornaviral 3Cs, through their abilities to bind RNA and act as a protease, to hijack host cell metabolism and defenses.

## 3. The 3C Protease Function Intervenes in Host Cell Processes

**Figure 4:**
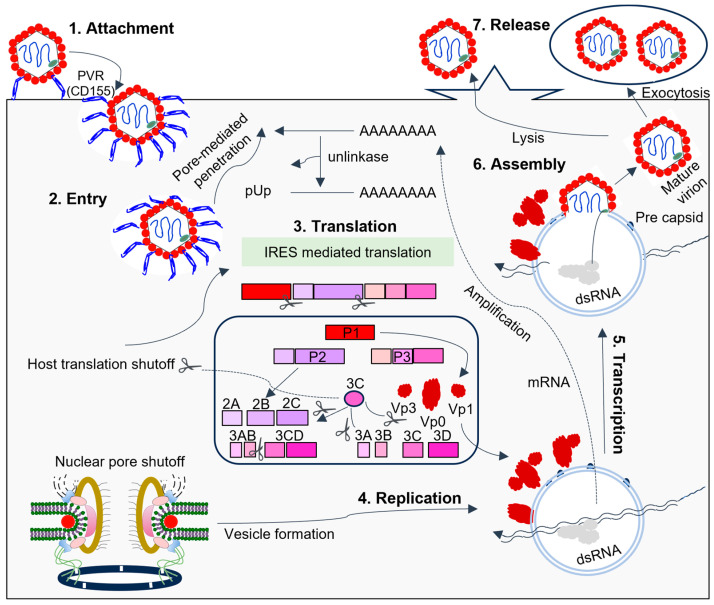
The picornavirus life cycle involves 3C interfering with host components to facilitate RNA replication. Endocytosis, promoted by the attachment of the virus to host receptors, allows the virus to enter the host cell. In PV, the receptor is CD155. Viral proteases cleave translation initiation factors, halting cellular cap-dependent translation. Replication occurs in endoplasmic reticulum-derived membrane vesicles in viral factories, using genomic single-stranded positive-strand RNA (ssRNA(+)) to create a double-stranded RNA intermediate. Transcription and replication produce viral mRNAs and new ssRNA(+) genomes. The packaging of genomic RNA into prepared procapsids leads to cell death and virus release. During SVV infection, the virion first attaches itself to the host cell ANXTR1 receptor before internalizing itself through an endocytic pathway [92,93]. Unlike other picornaviruses, SVV has a distinct uncoating mechanism and binding mode with its receptor.

The 3C protease cleaves the viral polyprotein and host cell proteins and also binds RNA which is essential for virus replication and inhibiting host cell responses [1,3]. Viral infection induces interferon (IFN) production inside the host cells owing to the antiviral immune response [87]. The co-evolution of viruses and host cells has led to the development of IFN-evasion techniques, allowing viruses to persist inside host cells, in which 3C plays an important role [69,87]. For instance, 3C plays a key role in blocking host signaling pathways, thus allowing the virus to circumvent host cell defenses and readily multiply inside host cells [88,89]. Picornaviruses inhibit antiviral protein synthesis and transport and block immune responses through transcription and cap-dependent translation [90,91]. Here, we discuss the multiple roles that 3C plays in the host cells, including halting transcription, inhibiting protein synthesis, blocking nucleocytoplasmic transport, and inducing cell death (see Figure 4).

### 3.1. Proteolysis by 3C Proteases Suppresses the Host Transcription and Translation Mechanisms

3C proteins play crucial roles in suppressing the host transcription and translation mechanism, such that energy and resources are redirected towards virus replication. The picornavirus enters the host cell, followed by the release of the picornaviral genome into the cytoplasmic matrix, where it can interact with ribosomes [89,94,95,96]. The 3C protein acts to suppress transcription and cap-dependent translation of host genes [94,97]. For instance, the 3C-mediated cleavage of histone H3 at its N-terminus generates a novel polypeptide Pi in BHK cells, which persists in association with chromatin [98]. This process provides binding sites for transcription [99,100]. Therefore, the host cell transcription may be halted by the cleavage of H3 into Pi [98,101]. 3C also proteolyzes transcription factors, including IIIC, cAMP response element-binding protein-1 (CREB1), octamer binding protein-1 (OCT1 or POU2F1, p53 (TP53), TATA-binding protein-associated factor 110 (TAF110 or TAF1C), and TATA box-binding protein (TBP), resulting in the blockage of transcription initiation by host RNA polymerases [102,103,104,105,106,107]. Recent evidence also shows that EV71 3C can cleave the cellular CstF-64 protein, which subsequently halts host RNA processing and polyadenylation [108]. SVV 3C cleaves MAV, TRIF, and TANK which blocks transcription [92,109,110].

**Figure 5:**
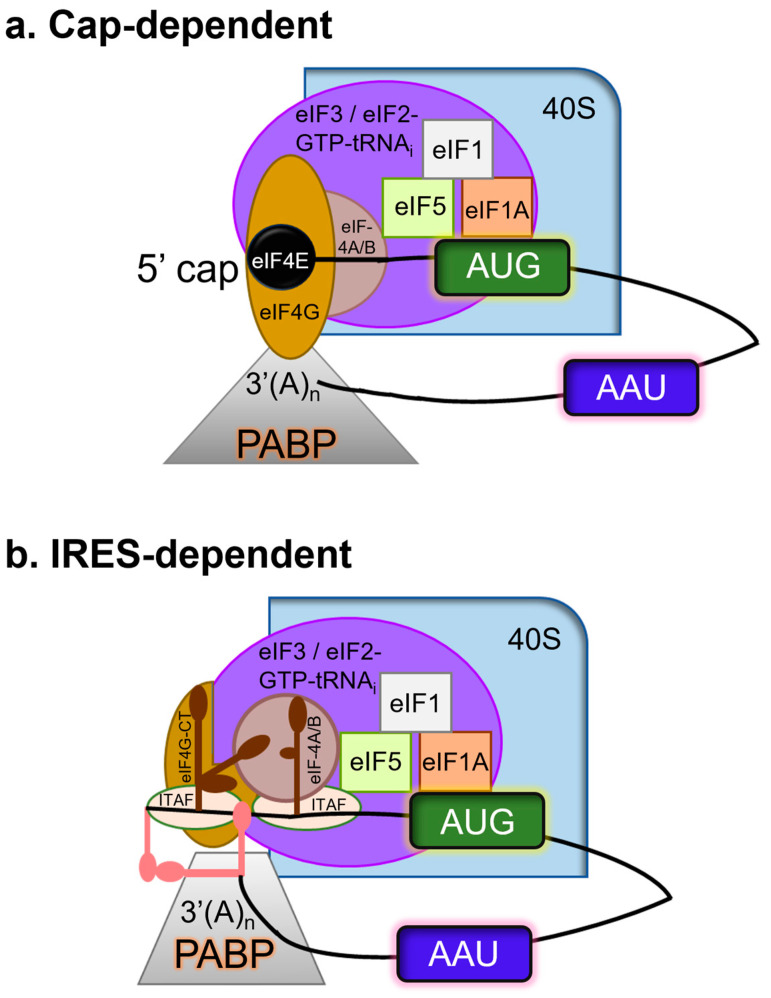
Translation initiation can be (**a**) cap-dependent and/or (**b**) IRES-dependent. Using the color code, only the primary factors mentioned in the text have been depicted for simplicity. Cellular mRNAs functionally pseudo-circularize as a result of the interaction between eIF4G and PABP. Regarding the FMDV genome, the 5′-3′ long-range communication among the 3′-UTR with the S fragment (a large stem-loop formed by the folding of the terminal structure at the 5′ end), in addition to 3′ UTR-IRES interaction, permits a comparable circumstance that is probably stabilized by binding factors for RNA. IRES-driven translational initiation requires a few eIFs and IRES-transacting factors (ITAFs), i.e., PCBP2, PTB, and Germin 5 which modulate IRES activity. 3C is crucial because it guarantees the internal initiation of translation by cleaving certain eIFs and ITAFs.

Picornaviruses inhibit host translation (see Table 1) through various pathways, including IRES-dependent translation for viral protein synthesis [18,90,111] (Figure 5). Cap-dependent protein and DNA repair processes are shut off in infected host cells due to the fragmentation of cellular components [112]. 

Seneca Valley Virus 3C (SVV 3C) cleaves eukaryotic initiation factors (eIFs) eIF4AI and eIF4G1, while PV, CVB, and HRV 3C proteases cleave eIF5B [18,58,63,93,113]. eIF4AI inhibition alters eIF4AII protein expression [114]. eIF5B cleaves at one site (VVEQ↓G), cutting the C-terminal and N-terminal regions from the conserved GTPase domain during infection processes [59,115]. These cleavage events also affect the termination of viral translation, leading to increased RNA packaging into new viral particles. The 3C protease cleaves the viral RNA-binding protein PCBP2, causing it to lose its K-homologous domain (KH3) [62]. This 3C-processed cleavage is also observed in HAV-infected cells [116]. The polypyrimidine tract-binding protein (PTB), including PTBP1 and PTBP2, is also cleaved by HAV 3C to inhibit viral translation and improve viral genome replication (and is also observed for PV 3C) [115,117]. The FMDV and EV71 3C proteases can cleave the Sam68 nuclear RNA-binding protein, allowing it to play nonnuclear functions in the cytoplasm [59,64,118,119]. Sam68 can engage with IRES in FMDV during infection and before viral RNA translation [64]. 

An indirect way of promoting virus protein translation is through the 3C-directed cleavage of nuclear pore proteins. Picornaviruses produce RNA in the cytoplasm, while cellular RNA is produced in the nucleus [120]. Picornaviral proteins target five nucleoporins (Nups; Nup62, Nup98, Nup 153, Nup 214, and Nup 358) to disrupt macromolecule trafficking via the nuclear pore complex (NPC) [121]. These proteins are specifically proteolyzed by 3C (e.g., as observed with HRV 3C) [3,88,122,123], demonstrating that picornaviruses change nucleocytoplasmic shuttling to meet nuclear-resident protein requirements but obstruct cellular mRNA export [124].

### 3.2. 3C-Promoted Apoptosis for Virus Release

Infected cells can exhibit 3C-induced apoptosis, as shown for SVV, HAV, and CVB [17,92,125,126]. When a picornavirus infects a cell, 3C causes cell death through both caspase-dependent and caspase-independent mechanisms, interferes with the cleavage of the Golgi apparatus and MAP-4, and controls the secretory pathway and intracellular membrane trafficking [49,127,128,129]. The 3C protein regulates apoptosis through various pathways, triggering Bax and cytochrome c secretion in mitochondria [130].

The cleavage of transcription and translation initiation factors induces the production of apoptotic bodies and DNA degradation, while also degrading the transcriptional activator p53 in vivo and in vitro [1,5,89,129,131]. PV–3C is essential for p53 degradation in cells infected with PV, promoting apoptosis through transcription-independent mechanisms [5]. CVB-3C activates caspase-9-based apoptosis through caspase 3 activation [127]. 3C is essential for SVV-mediated apoptosis following caspase 3, while HAV 3C causes cell death without the aid of caspase [93,127]. VAD–fmk caspase 1 and DEVD–fmk inhibitors interfere with 3C-induced apoptosis, suggesting 3C initiates caspase cascades during late PV infection [132]. The 3C-induced apoptosis in EV 71 is related to its proteolytic activity as reported in several studies [133].

### 3.3. 3C Interferes with Host Cell Defenses

Host cells utilize a variety of antiviral mechanisms to combat picornavirus infection, including stress granule formation, innate inflammatory responses, autophagy, and a few others [2,125,134]. Picornaviruses must attack the cellular proteins engaged in these antiviral defenses to survive, and 3C is essential to these processes [87,88,89]. 

When an organism is infected with a picornavirus, 3C triggers the host innate immune system to employ host pattern recognition receptors (PRRs) to identify the presence of pathogen-associated molecular patterns [87]. These include retinoic-acid-induced gene-I (RIG-I), cytosolic RIG-like RNA helicases such as melanoma differentiation-associated gene (MDA-5), and transmembrane PRRs such as Toll-like receptors (TLRs) [135,136]. PRRs enlist various particular adaptor proteins to initiate a signaling cascade downstream and stimulate three primary pathways for the production of IFNs: the IFN regulatory factor (IRF) pathway, the mitogen-activated protein kinase (MAPK) pathway, and the nuclear factor kappa-light-chain-enhancer of activated B cells (NF-κB) pathway [137]. IFNs have the ability to trigger hundreds of interferon-stimulated gene factors (ISGs) that strengthen host defenses through autocrine or paracrine signaling [138]. Table 2 summarizes the key signaling mechanisms that inhibit the generation of IFNs from picornavirus 3Cs.

Through its caspase-dependent protease activity, 3C degrades STAT1, STAT2, IRF9, and karyopherin1, thus inhibiting IFN-α signaling in SVV [92]. It was also discovered that 3C cleaves STAT2 at Gln758 in the transactivation domain. The cleaved products of STAT2 were found to reduce its capacity to trigger the production of IFN-stimulated genes and activate IFN-stimulated response element activity [92]. Furthermore, 3C was found to hamper the IFN-stimulated gene factor 3 complex nuclear import and formation [87]. Lastly, 3C was found to cause the degradation of karyopherin 1 to prevent STAT1/STAT2 nuclear localization. When combined, SVV 3C can disrupt the type I IFN response by focusing on STAT1–STAT2–IRF9 and karyopherin α1 signals [92,109]. This reveals a new way that SVV uses to avoid host defenses. Other viruses may operate through similar mechanisms, although this would require further research. 

The recognition of viral infections in the host cell initiates the antiviral response. Double-stranded RNA, or dsRNA, is a vital ligand for 3C of picornaviruses, as it triggers the cellular immune response [143]. Cytosolic dsRNA can be sensed by RIG-I-like receptors (RLRs), which include melanoma differentiation-associated protein 5 (MDA5) [144,145,146]. The interferon regulatory factor (IRF) family of transcription factors is nuclear translocated as a result of a signaling cascade that is triggered when MDA5 binds to dsRNA [147]. Nuclear IRFs can cause the expression of antiviral interferon-stimulated genes (ISGs) in neighboring uninfected cells [148]. They can also induce the transcription of several genes with antiviral functions, such as IFIT1 and RSAD2, as well as proinflammatory cytokines, such as IFNs, for which the protein products are secreted [143].

## 4. RNA Binding and the Viral RNA Template Transformation

The 3C protein is a key factor for the formation of protein-RNA complexes that act toward viral replication and release from host cells. The virus requires a way to transition between RNA replication and translation [149]. Viral RNA exists in numerous copies that are manipulable on their own [150]. The two events may take place concurrently in the host cell utilizing different copies of identical RNA template. The 5′-NTR of the picornaviral genome contains cis-acting replication elements (CRE), composed of oriL (left), oriI (internal), and oriR (right) [18,151]. These three CREs are all thought to interact with 3C (or 3CD), which may also impact protease activity and/or its interactions with replication membranes [47]. It has been shown that the conformational energy landscape of 3C is altered by RNA and peptide binding, potentially leading to different protein-RNA complex sites [49]. Earlier research from our lab shows that the structural dynamics of 3C are altered by RNA/peptide binding, which has an impact on both the binding site and predicted binding site of the other ligand [47]. The conformational dynamics on the pico-to-nanosecond and micro-to-millisecond timescales are also altered by RNA binding. It has been suggested that RNA binding selects different conformations, influencing peptide interaction with 3C and that the higher energy conformation may be crucial for interacting with protein substrates. These actions may also be important for 3C contribution towards switching between translation and RNA replication processes, which must use the same RNA template [149].

Picornaviral RNA serves as a template for translation and RNA replication, enabling IRES-driven viral protein generation [66]. A complementary negative-sense RNA molecule is produced using the same template, leading to the replicative form [88,152]. This intermediate produces a multiple-stranded RNA complex, generating positive-sense RNA molecules [69,119,153]. These RNA molecules are either recycled or assembled into offspring virions, using nuclear proteins found in uninfected cells. The 3C from PV, FMDV, EMCV, CVB3, and EV71 can induce the cleavage of Ras GTPase-activating protein-binding protein 1 (G3BP), which is also crucial for innate immune responses to DNA viruses [65,154,155]. As such, cleaving G3BP1 may increase the risk of secondary DNA viral infections, promoting viral replication. During HAV, CVB3, SVV, and EV-A71 infection, 3C-induced cleavage of Toll/IL-1 receptor-domain-containing adapter-inducing interferon (TRIF) is significant [110,156,157]. Picornaviruses and host cells both possess translational regulators, and 3C protease can initiate viral RNA replication by cleaving translation-associated proteins linked to viral RNA [18,93].

## 5. Roles of 3C(D) and Other 3C-Containing Polyproteins in Picornavirus RNA Replication

The 3C protein also exists as a domain in the 3CD precursor protein. As noted, 3C plays crucial functions in the RNA replication complex assembly and the cleavage of the viral polyprotein during virus replication [1,20,21,138]. Several RNA replication components, including the 5′ UTR, CREs, and the 3′ UTR, are necessary to produce newly isolated picornavirus RNA [15]. Several studies are in agreement with the PV RNA synthesis-initiating model [158,159,160,161,162]. In this model, 3CD plays a significant role by attaching to the stem-loop d of the 5′ UTR cloverleaf, enhancing the binding of the poly(rC)-binding protein 2 (PCBP2) to the stem-loop b [163,164].

The RNA-binding ability of the RNA in 3C and 3CD is crucial for efficient uridylylation of VPg [3,165,166]. HRV-14 3C binds to the stem-loop d of the cloverleaf, while HAV 3C, 3CD, and 3ABC show efficient binding to both the 5′ and 3′ UTR [127,152,167,168,169,170,171]. 3ABC has a stronger binding capacity, while 3CD has a weaker RNA-binding ability [8]. In the absence of both 3C and 3CD, 3B_3_3C and 3B_123_3C serve as alternative substrates for uridylylation [172]. In cases where FMDV 3C substitutes 3CD in VPg uridylylation, the efficiency is reduced.

The 3CD cloverleaf complex incorporates 3AB in the replication process, which is cleaved by either 3AB or VPg, releasing the 3D enzyme with polymerase activity [173,174]. This enables the uridylylation of VPg and interactions with cre, making it a template for VPg uridylylation [9,67,165]. The complex formation is completed when 3AB–3CD binds to the 3′ UTR, and PABP—post-cleavage by 3CD—associates with the poly(A) tail, setting the stage for new RNA strand synthesis [161,175].

The functional differences between 3C, 3D, and 3CD may be due to differences in protein conformational dynamics [25,48]. We conducted NMR experiments to test these dynamics across multiple timescales [25]. The results identified differences in conformational dynamics in crucial regions such as enzyme active sites and RNA and lipid binding sites. The differences in conformational dynamics near the active site and RNA binding site may help to differentiate the function between 3C and 3CD [25]. Differences in conformational dynamics may also help to explain allosteric communication between the 3C and 3D domains of 3CD. The expansion of the conformational ensemble in 3CD may enable it to perform additional functions not achievable in 3C and 3D alone.

## 6. 3C Protease Inhibitors

**Figure 6:**
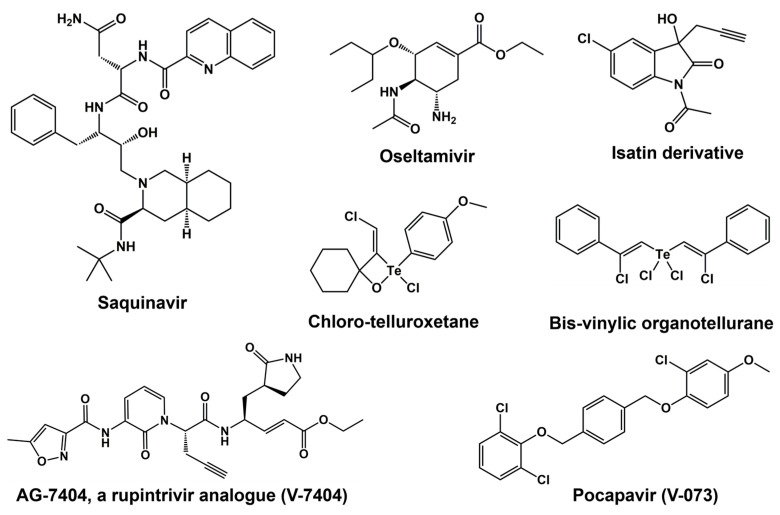
Chemical structure of various 3C protease inhibitors.

Drug–protein interactions can be better understood at the molecular level through computational and bioinformatics tools, which can be used to expedite the design and development of enhanced antivirals targeting important proteins [176]. A specific field of structure-based/ligand-based drug design called computer-aided drug discovery (CADD) boasts several successes, including in the field of antivirals. The development of FDA-approved medications such as Saquinavir (anti-HIV) and Oseltamivir (anti-influenza virus) has confirmed the effectiveness of these methods [176] (see Figure 6 for chemical structures). One intriguing family of flexible lead compounds among the various peptidomimetic and small-molecule inhibitors that have been tried against viral proteins is isatin and its derivatives. Isatins have demonstrated antiviral efficacy against a variety of picornaviruses through interactions with 3C proteases.

Initial reports described the usage of tellurium compounds as PV 3C protease inhibitors. PV 3C was found to be rapidly inactivated by a stoichiometric and covalent reaction with both chloro-telluroxetane and bis-vinylic organotellurane [177]. Additionally, these substances have second-order rate constants for inhibition of human cathepsins B, L, S, and K that are greater than those found for PV 3C. Under low micromolar concentrations and below the lethal threshold for host cells, chloro-telluroxetane prevents the replication of PV in human embryonic rhabdomyosarcoma cells [177]. Although bis-vinylic organotellurane is a more potent antiviral drug, at 10 μM it inhibits cell viability by 20%, a dose that almost stops virus growth. This is the first account of this family of drugs antiviral properties through suppression of viral 3C proteinase.

Most clinical compounds have been rejected because of their weak selectivity between antiviral activity and cytotoxicity or their lack of potency [178]. Two compounds have been found so far in the results that satisfy the requirements for being considered poliovirus antiviral development candidates. One such substance is pocapavir (V-073), a capsid inhibitor licensed by ViroDefense Inc. and initially discovered by Schering-Plough (SCH 48973). The second substance is the 3C protease inhibitor AG7404, a rupintrivir analogue that was found by Agouron (Pfizer Inc., McPherson, KS, USA) and is currently being developed as V-7404 by ViroDefense Inc [138]. Initially created by Pfizer as an anti-rhinovirus drug, the 3C protease inhibitor V-7404 also exhibits strong in vitro anti-poliovirus action [179,180]. Although V-7404 is being positioned for combination treatment with pocapavir if drug resistance arises when treating PID patients with a single compound, pocapavir is currently being developed as a single-agent treatment. The Task Force for Childhood Development and Survival has entered into a contract with ViroDefense Inc. to assess the PV antiviral candidate V-073. In addition, ViroDefense Inc. is taking part in an NIAID program to provide specific preclinical services.

V-7404 is an orally accessible, single-dose organic compound which inhibits the human rhinovirus (HRV) 3C protease irreversibly [180]. The concentration-dependent suppression of HRV 3C-mediated polyprotein processing in infected cells by this compound directly confirms that the inhibition of 3C protease is the cause of the cell-based antiviral action. Nonclinical safety investigations conducted in vitro and in vivo have demonstrated that V-7404 is safe at the highest doses that could be administered [181]. In a phase-I-ascending single-dose research study, oral dosages of Compound 1 up to 2000 mg have been shown to be safe and well tolerated in healthy individuals.

## 7. Conclusions

Positive-strand RNA viruses act to maximize their genomic information content in part by encoding multifunctional proteins, as exemplified by 3C. Picornaviral 3C performs important roles in viral replication and inhibition of host cell responses through its ability to interact with RNA and act as the main protease. It is noted that many of these functions may be enacted through its polyprotein precursors (e.g., 3CD). The RNA-binding ability of 3C is crucial for initiating viral RNA synthesis. The mode and interaction specificity of 3C for binding various RNA is still under investigation.

The 3C protease intervenes in host cell transcription, translation, and replication events. It also plays critical roles in apoptosis and generation of phosphatidylserine (PS) lipid-rich vesicles that assist in virus release and spread. The binding of 3C to RNA alters the structural dynamics of 3C and controls how 3C functions with other cellular substrates. The cellular substrates of 3C have varying activities in host cells, potentially advantageous to viruses at different lifecycles [1,17]. Several newly identified substrates for PV and CVB3 3C have been found employing terminal amine isotopic labelling of substrates [182]. High-throughput technologies may help discover new cellular substrates and better understand how 3C modifies host processes and pathophysiology [183].

Picornaviruses inhibit antiviral protein synthesis and transport, suppressing immune responses through transcription and cap-dependent translation, and using mechanisms such as PARP9–DTX3L complex to regulate ISG expression and the immune response [6,24,77,83,128]. Furthermore, 3C precursors are also crucial in viral replication owing to their ability to hijack cellular resources and establish an optimal intracellular condition for the virus life cycle. The 3C-mediated cleavage of PABP disrupts mRNA circularization, and 3C also alters specific cellular components and fragments, such as eIF4G, PTB, and PCBP2 [66,184,185,186,187]. These findings highlight the critical functions that 3C performs in viral replication by ensuring translation, replication, and the transition from translation to replication.

Given the central role of 3C (or its precursors) in the many aspects of the picornaviral life cycle, there has been much effort in the development of 3C inhibitors [188,189]. To date, there are only a few treatments available for picornavirus infection [190,191]. The development of new antiviral treatments may become more accessible with a broader understanding of the roles played by picornavirus 3C. Efforts are currently ongoing to identify irreversible inhibitors for 3C, with further investigations into its molecular functions, structure, and dynamics potentially aiding in this goal [176,192].

## Figures and Tables

**Table 1:** The 3C cleavage of proteins involved in cellular translation. Cleavage site identified by underline.

Virus	Protein from the Host Cell	Activity	Cleaving Site	Reference(s)
Poliovirus (PV)	eIF5B	Inserts the initiation tRNA for methionine on the start codon of mRNA	LeuCysAlaAlaValGluValMetGluGln^478^GlyValProGluLysGluGluThr	De Bryne S et al. (2008) [59]
	PCBP2	Regulation of gene expression through translational activation	AlaMetGlnGln^253^SerHisPhePro… IleGlyArgGln306GlyAlaLys	Perera R et al. (2007) [60]
	PABP	Involved in the start of the translation and the shortening of poly (A)	Not certain	Kuyumcu-Martinez NM et al. (2004) [61]
Foot-and-mouth disease virus (FMDV)	eIF4A I	Unwinds double-stranded RNA and allows the ribosomal subunit 40S to bind capped mRNA	CysIleGlyGlyThrAsnValArgAlaGlu^143^ValGlnLysLeuGlnMetGluAla	Li W et al. (2001) [62]
	eIF4G I	Transports mRNA to the 40S ribosome to initiate translation	ArgArgSerGlnGlnGlyProArgLysGlu^712^ProArgLysIleIleAlaThrValLeu	Belsham GJ et al. (2000) [63]
	Sam68	Responsible for cell growth and division	C-terminal region	Lawrence P et al. (2012) [64]
Coxsackievirus (CVB)	eIF5B	Inserts the initiation tRNA for methionine on the start codon of mRNA	LeuCysAlaAlaValGluValMetGluGln^478^GlyValProGluLysGluGluThr	De Bryne S et al. (2008) [59]
	G3BP1	Ras–GAP-interacting RNA-binding protein	GluAlaGlyGluGln^325^GlyAspIleGluPro	Fung G et al. (2013) [65]
Human rhinovirus (HRV)	eIF5B	Inserts the initiation tRNA for methionine on the start codon of mRNA	LeuCysAlaAlaValGluValMetGluGln^478^GlyValProGluLysGluGluThr	De Bryne S et al. (2008) [59]
Hepatitis A virus (HAV)	PCBP2	Regulation of gene expression through translational activation	IleGlyArgGln^306^GlyAlaLysIle (Postulated)	Zhang B et al. (2007) [66]
	PABP	Involved in the start of the translation and the shortening of poly (A)	Not certain	Zhang B et al. (2007) [67]
Encephalomyocarditis virus (EMCV)	PABP	Involved in the start of the translation and the shortening of poly (A)	ValArgProProAlaAlaIleGln^437^GlyValGlnAlaGlyAla	Mariko K et al. (2012) [68]
Seneca valley virus (SVV)	TRIP MAVS TANK	Suppresses host type-I interferon production	Glu-Gln or, Gln-Gly	Qian S et al. (2017) [69]

**Table 2:** Key signaling mechanisms in picornaviruses that inhibit the generation of IFNs from 3C.

Virus	Signaling Pathways	IFN Type	References
EV71	Inhibits IRF7 and IRF9	Type I IFNs	Lei X et al. 2010 [139]
EMCV	Cleaves TANK and inhibits TRAF6-mediated NF-κB signaling	Type I IFNs	Huang L et al. 2015 [137]
CVB3	Cleaves MAVS and TRIF	Type I IFNs	Mukherjee A et al. 2011 [140]
CV–A6, EV–D68	Cleaves TAK1 to inhibit NF-κB signalling	Not clear	Yajuan R et al. 2017 [135]
FMDV	Cleaving TANK and NEMO	Not clear	Zhao T et al. 2007 [141]
HAV	Cleaving MAVS and NEMO	Type I IFNs	Yang Y et al. 2007 [142]
SVV	Cleaving MAVS, TRIF, and TANK	Type I IFNs	Qian S et al. 2017 [69]

## Data Availability

Not applicable.
